# Health of Dentists

**Published:** 1869-06

**Authors:** 


### HEALTH OF DENTISTS.

There can be no doubt but the Dental practitioner encounters a full share of the dangers that environ the pathway of human life and health, under all circumstances, conditions and avocations, modified by the peculiarities of his own individuality and special surroundings.

To avoid contact with these adverse influences, is perhaps utterly and absolutely impracticable, so far as the present race of mankind is concerned; most assuredly human ingenuity, and the most persistent effort, has never approximated the attainment of this desirable end.

Men may sometimes avoid contact with the special danger that threatens their present condition or course of life, simply by hurling themselves against, or upon other dangers equally formidable, and for which they are equally unprepared. As yet, nothing better has been devised, than to fortify the system in the highest attainable condition of health, thus to reserve a fund of *life power* ready to oppose, whatever extraordinary assault external influences may make on our physical organization.

To illustrate, we will suppose that our "mutual friend," ———, who is endowed by nature with an extraordinary share of sympathetic impressibility, if he expose himself to sympathetic contact while he is in vigorous health, one of the predominant traits, or elements of his organic nature, is thus brought into healthy activity; under favorable circumstances a salutary reaction follows, imparting health and vigor to the entire system.

Whereas, if his general health be impaired,—if his general nervous system be in a feeble or irritable condition, the same sympathetic contact would aggravate the irritability, or increase the prostration, and the general constitutional result would be injurious and painful, rather than agreeable and salutary.

If he avoid sympathetic contact altogether, derangement necessarily supervenes from leaving an important feature, a predominant element of his organism in neglected idleness, to seek activity in misdirected pursuit of improper objects.

The individual, endowed with extraordinary gastric powers, will suffer more and die sooner from deficiency or destitution of food, than one whose digestive and nutritive system, bears a more harmonious or symmetrical proportion to his muscular, mental, and sympathetic constitution.

There was no departure from the true doctrine of human character, in the story of an Arkansas ruffian, who was reported to have been found on board one of the river steamers coiled up in a salt-bin, and when called upon for an explanation, declared that he was a "ring-tailed roarer," that he could whip his weight in wild cats, but not having had a fight for three days, he was afraid he would spoil.

Predominant belligerent combativeness, prompts the unfortunate possessor to seek fame and fortune in personal contests; innate self-consciousness, clamorous for its wonted indulgence, its accustomed exercise.

I can not now speak of the expediency of yielding to the influence of untoward, or wayward inclinations or impulses; but would remark that the greater the deviation from symmetry of character, the stronger the tendency to seek special gratification, and hence arises the obliquity of inclination or impulse, that too often lead to universal and utter derangement.

The principles set forth, and the advice tendered in the article on preserving the health of Dentists, proposes to point out the way to avoid these dangerous errors.

How to correct these aberrations, incurred or established, is quite another matter, and may serve to furnish the subject matter for a future article.

